# Esophageal Cancer With Cardiac Metastasis: Approach to Cardiac Masses in Patients With a Known Malignancy

**DOI:** 10.7759/cureus.64486

**Published:** 2024-07-13

**Authors:** Hadassah Stein, Matthew Bellesheim, Marc Ganz, Daniel Miller, Salman Syed, Bradley Minter

**Affiliations:** 1 Radiology, Northwell Health, New York, USA; 2 Internal Medicine, Northwell Health, New York, USA; 3 Urology, State University of New York Downstate Health Sciences University, New York, USA; 4 Internal Medicine, Icahn School of Medicine at Mount Sinai, Queens Hospital Center, New York, USA

**Keywords:** lymphatic spread, echocardiography, diagnostic imaging, esophageal cancer, cardiac metastasis

## Abstract

Cardiac metastases occur in a significant proportion of cancer patients and profoundly affect clinical outcomes and management strategies, especially in esophageal cancer, where the metastasis typically targets the left heart due to its unique lymphatic spread. Diagnostic imaging is crucial for patients with cardiac symptoms or electrocardiogram (EKG) changes, as it significantly influences treatment decisions, including the operability of the primary tumor and the risks associated with left-sided metastasis, such as the potential for embolization leading to stroke. This case report provides a detailed analysis of esophageal cancer metastasizing to the left atrium, highlighting diagnostic challenges and discussing the appearance of cardiac metastasis across various imaging modalities. The report examines the advantages and limitations of each imaging technique, offering insights into their roles in accurate diagnosis and effective management in complex clinical scenarios.

## Introduction

A cardiac metastasis is far more prevalent than primary cardiac tumors, occurring in up to 18% of cancer patients [1]. The majority of these cases involve malignant melanoma and primary mediastinal tumors, although metastases from the breast, esophagus, thyroid, and lung cancers are also common [2,3]. Typically, patients are asymptomatic, but some may develop serious cardiac complications such as congestive heart failure, valvular disease, pericardial effusion, dysrhythmia, syncope, embolism, or cardiomegaly [4,5]. In patients presenting with symptoms, or those with EKG changes or CT findings that suggest cardiac involvement, an echocardiogram should be promptly conducted due to its implications for management, especially since confirmed metastatic disease to the heart might preclude surgical options [6].

The pathways for tumor spread to the heart include direct invasion, hematogenous routes, lymphatic channels, or intracavitary diffusion via the inferior vena cava or pulmonary veins [4,7]. The type of cardiac structure affected often suggests the likely path of invasion; for instance, lymphatic spread is typically associated with pericardial involvement, while hematogenous routes are suggested in myocardial invasion [2], with lymphatic spread being the most common overall [6]. Endocardial metastases are rare and usually result from hematogenous spread that leads to intracavitary lodging, though they can also stem from myocardial metastases [7]. Additionally, many mediastinal and intrathoracic tumors metastasize through direct extension due to their proximity to the heart [3].

The right heart is more frequently affected by cardiac metastasis than the left, likely due to the lower flow velocity which facilitates cancer cell seeding and colonization [3]. This contrasts with primary cardiac tumors that mainly affect the left atrium and potentially block flow into the left ventricle [2]. In the case of metastatic esophageal cancer discussed here, the metastasis involved the left heart, which is explained by the unique route of lymphatic spread characteristic of esophageal cancers [8].

Normally, lymph drains from the posterior wall of the heart, where the left atrium resides, into the carinal and parasternal lymph nodes. This lymphatic connection provides a channel for tumor cells to initially infiltrate the mediastinal lymph nodes and subsequently access the pericardium and left-sided myocardium through reverse lymphatic flow, which can be promoted by tumor-induced lymphatic obstruction, increased intraluminal pressure, and inflammatory changes [8,9]. 

Overall, cardiac metastasis signifies a poor prognosis as it generally indicates extensive metastatic disease [5]. Currently, there are no established guidelines for the management of cardiac metastasis. However, the available treatments typically include palliative surgery or chemoradiation [3,5]. Special consideration for surgical resection is warranted in cases of left-sided metastasis due to the risk of emboli and subsequent stroke [10]. We present the following case that highlights the nuances of cardiac metastasis in a patient with metastatic esophageal cancer, illustrating the real-world implications and challenges of managing such complex cases

## Case presentation

A 61-year-old male presented with a past medical history of atrial fibrillation, hypertension, and metastatic gastroesophageal adenocarcinoma, being treated with multiple chemotherapy regimens, most recently Paclitaxel and Ramucirumab. He developed retrosternal pain following an esophageal stenting procedure performed to manage worsening dysphagia. The pain was described as intermittent, non-radiating, and moderate in intensity, ranging from 4-5/10 on the pain scale, and persisted for 1-2 days. The discomfort worsened when the patient was lying down or making positional changes, but it somewhat alleviated when he bent forward. He did not report any other symptoms, such as syncope, dyspnea, chest pain, cough, or peripheral edema. The ongoing medications at the time of presentation included Rivaroxaban 20 mg daily and metoprolol, which had been prescribed three years prior for paroxysmal atrial fibrillation.

**Figure 1:**
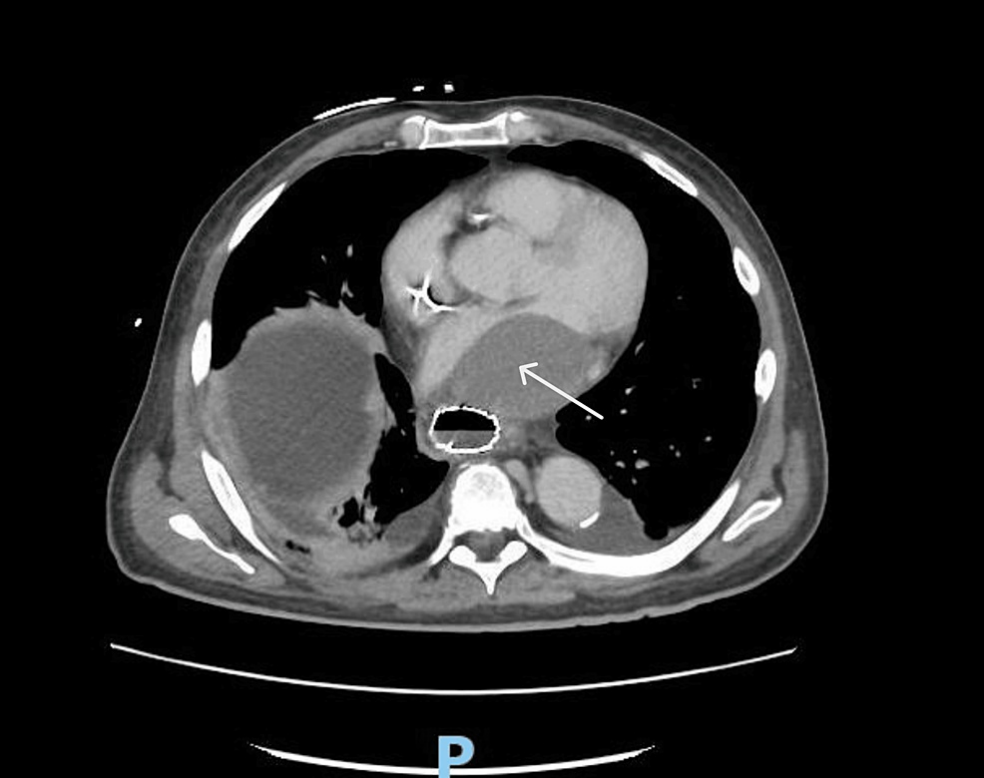
CT abdomen/pelvis with IV contrast with an ovoid thrombus versus mass/metastasis visualized in the left atrium

During an evaluation, an EKG showed atrial fibrillation with rapid ventricular response at a rate of 143 beats per minute, low voltage QRS, and a persistent right bundle branch block previously identified on earlier EKGs. Imaging studies, including a chest radiograph, revealed a right-sided chemoport and mild right-sided atelectasis. More revealing, however, was a CT scan of the abdomen and pelvis which indicated the presence of an ovoid 4.3 x 6 x 5 cm mass in the left atrium, raising differential diagnoses of thrombus, tumor, or metastasis, alongside an esophageal mass at the gastroesophageal junction that appeared inflamed or possibly involved with the stenting (Figure 1). 

**Figure 2:**
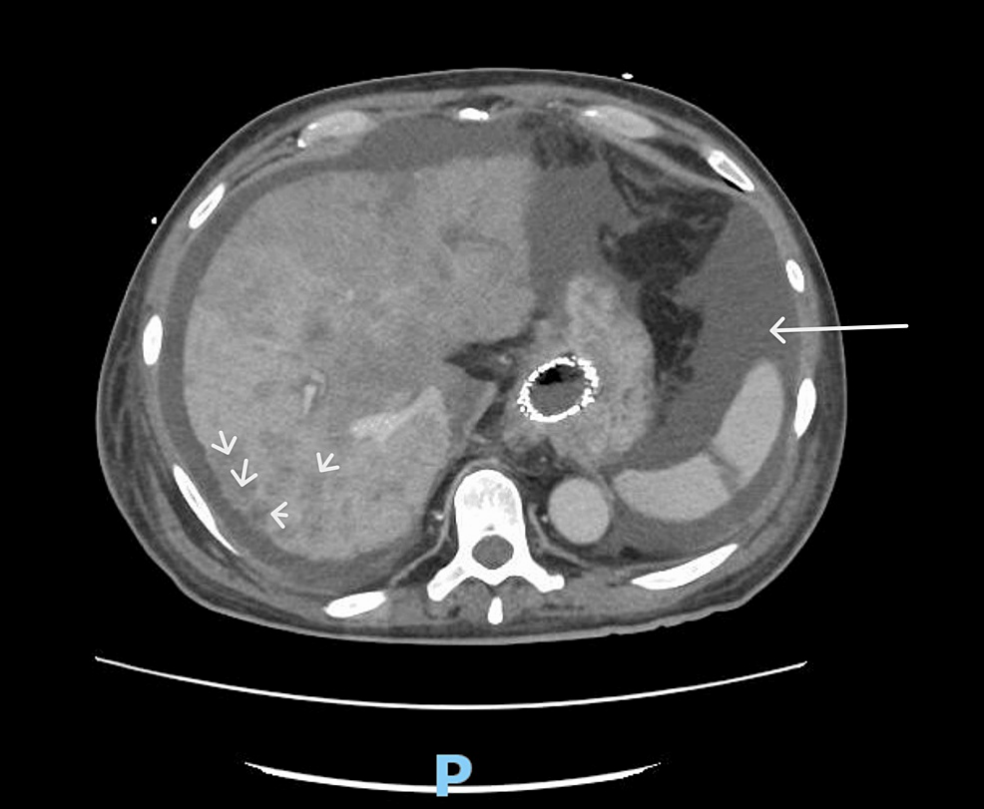
CT Abdomen with IV contrast involving enlarged spleen and multiple hepatic lesions

This scan also noted mild bilateral pleural effusions and scattered atelectasis across various lung segments, extensive low-density lesions throughout the liver suggestive of metastatic disease, and an enlarged spleen with a localized area of infarction (Figure 2). Notably, enlarged precaval lymph nodes up to 1.8 cm and scattered subcentimeter mesenteric lymph nodes were observed, indicating possible lymphatic involvement. 

A subsequent transthoracic echocardiogram (TTE) provided further insights, revealing a mildly dilated left atrium with a large, ovoid mass characterized by cystic and echogenic components, measuring 6.6 x 4.7 cm and predominantly occupying the left atrial cavity. The mass was attached to the posterior left atrial wall, and its imaging characteristics suggested a neoplastic origin, though the possibility of a thrombus could not be definitively excluded. Systolic function remained normal.

Further diagnostic modalities such as a dedicated transesophageal echocardiogram (TEE) and cardiac MRI were considered. However, after detailed discussions regarding the patient's overall prognosis, therapeutic goals, and the unlikely influence of these tests on management strategies, a decision was made against pursuing these additional invasive procedures. Reflecting on his quality of life and the advanced stage of his disease, the patient opted for a palliative approach focusing on comfort care.

## Discussion

It is essential to understand the presentation of cardiac metastasis across various imaging modalities, including CT, MRI, PET, and ultrasound, which are often utilized in cases where there is already a high suspicion of cardiac metastasis. These modalities may also reveal cardiac metastasis incidentally on scans performed for other reasons. On non-contrast CT, cardiac metastasis can appear as a hypodense lesion or as a filling defect on contrast-enhanced CT [11]. CT is particularly effective due to its large field of view and excellent spatial resolution, facilitating the detection of pericardial effusions and cardiac masses. Additionally, calcifications, which are significant in assessing cardiac metastasis from calcifying primary tumors like osteosarcoma, are easily detected on CT [12]. PET scans are valuable for identifying and monitoring metastatic disease, often showing increased radiotracer uptake localized to the heart [13].

An echocardiogram remains the best initial test for evaluating cardiac metastasis and excluding other differential diagnoses, such as mitral valve prolapse, foreign bodies, vegetations on valves, and intracavitary thrombi. Vegetations typically attach to valve leaflets, potentially leading to complications like chordal rupture or leaflet perforation, which can result in leaflet flailing or regurgitation. Atrial thrombi, often associated with atrial fibrillation, appear highly echogenic and can be found in the left atrial appendage or as free-floating formations, while ventricular thrombi typically occur with ventricular aneurysms [6,12]. Myxomas, the most prevalent primary cardiac tumor, usually affect the left atrium, attaching to the interatrial septum and exhibiting mobility [6]. Primary cardiac fibromas are known for their characteristic central calcifications that produce strong echoes and acoustic shadowing [11]. If an enhancing agent is used, myxomas display some diffusion but less so than the nearby myocardium; in contrast, malignant tumors are highly vascular and show pronounced enhancement [14].

However, echocardiography has limitations, such as poor visualization of the heart's apex and base, a narrow field of view, and dependency on the operator's skill [12]. Notably, a TEE provides superior visualization of masses in the left atrium and pulmonary veins due to the proximity of the transducer to these structures [6].

Cardiac MRI is advantageous for differentiating various tissue types, aiding in distinguishing infiltrative metastasis from the myocardium, and differentiating tumors from thrombus. Gadolinium-enhanced MRI shows avid enhancement in highly vascularized metastases. The enhancement pattern also helps distinguish chronic thrombus, which may show peripheral enhancement due to blood entrapment in surface irregularities, from metastatic disease, which typically shows central enhancement. However, MRI's utility can be limited by its cost, access issues, and contraindications for some patients, such as those with certain types of defibrillators [12].

A biopsy of the cardiac mass, although definitive for diagnosis, is often not pursued due to perceived futility [14]. In this particular case, the imaging characteristics of the intracardiac mass strongly suggested a diagnosis of cardiac metastasis. Additionally, an incidental splenic infarction was identified, which might relate to an underlying hypercoagulable state due to malignancy. This is a common scenario in cancer patients that could also link to the cardiac mass, whether through embolism from cardiac metastasis or thrombus from atrial fibrillation [15].

## Conclusions

In conclusion, the case of cardiac metastasis involving the left heart in a patient with metastatic gastroesophageal adenocarcinoma underscores the importance of accurate diagnosis and tailored management in the complex interplay of cancer and cardiac involvement. It is essential for radiologists to adeptly differentiate cardiac masses on imaging since the diagnosis will significantly influence management strategies. For instance, while cardiac metastasis may occasionally warrant surgical resection, an atrial thrombus would typically necessitate anticoagulation therapy. Moreover, cancer's induction of a hypercoagulable state adds complexity, predisposing patients to both thrombotic and metastatic pathologies. As with all medical interventions, the benefits of any treatment must be carefully weighed against potential risks, ensuring alignment with the patient’s overall goals of care. Ultimately, even with the advancements in imaging and therapeutic modalities, the prognosis for cardiac metastasis often remains poor, highlighting the necessity for a focus on palliative care to enhance the quality of life for patients in the terminal stages of their disease.
